# The Effect of Diabetes and Hyperglycemia on Horizontal Guided Bone Regeneration: A Clinical Prospective Analysis

**DOI:** 10.3390/healthcare11121801

**Published:** 2023-06-19

**Authors:** Paolo De Angelis, Edoardo Rella, Paolo Francesco Manicone, Giuseppe De Rosa, Sofia Gallottini, Margherita Giorgia Liguori, Piero Papi, Viviana Desantis, Pier Carmine Passarelli, Antonio D’Addona

**Affiliations:** 1 Department of Head and Neck and Sensory Organs, Division of Oral Surgery and Implantology, Fondazione Policlinico Universitario A. Gemelli IRCCS—Università Cattolica del Sacro Cuore, 00168 Rome, Italy; 2 Department of Life, Health and Environmental Sciences, Università degli Studi dell’Aquila, 67100 L’Aquila, Italy; 3 Department of Oral and Maxillo-Facial Sciences, Sapienza University of Rome, 00168 Rome, Italy; 4 Department of Oral Surgery, University of Milan, 20122 Milan, Italy

**Keywords:** diabetes, bone regeneration, hyperglycemia

## Abstract

Guided bone regeneration (GBR) is a reconstructive procedure for treating atrophic alveolar ridges. This study aims to assess the correlation between different glycemic control levels and clinical findings in patients undergoing horizontal GBR before implant placement. The study population consisted of all patients requiring horizontal GBR. Patients were divided into three groups based on HbA1c levels: non-diabetic normoglycemic patients (HbA1c < 5.7%), non-diabetic hyperglycaemic patients (HbA1c < 6.5%), and patients with controlled diabetes (HbA1c < 7%). The primary outcomes were the horizontal (mm) and vertical (mm) dimensional changes of the alveolar ridge 6 months after the procedure. The study sample consisted of 54 patients. Sixty-eight implants (95.8%) were classified as “successful,” meaning the possibility of inserting a standard-sized implant following the GBR (diameter ≥ 4 mm). There was a statistically significant difference between the three groups in terms of horizontal gain at 6 months: in particular, there was a statistically significant difference between group 1 and group 2 (*p* = 0.026) and between group 1 and group 3 (*p* = 0.030). The present investigation showed that patients with HbA1c levels below 7% could undergo GBR and obtain a statistically significant horizontal bone gain.

## 1. Introduction

Diabetes is a major endocrine disorder characterized by an alteration in carbohydrates, lipids, and protein metabolism leading to an increased glucose concentration in the blood, called hyperglycemia. It affects about 10% of the population, but often, 2% to 3% of the population is misdiagnosed [1].

Diabetes can be classified into different categories, with the two main forms of the disease being commonly called Type 1 (juvenile diabetes) and Type 2 [2].

Diabetes can have different manifestations depending on whether diabetes is type 1 or 2, but most symptoms are similar [3]. Type I diabetes can cause loss of weight, polyphagia, polyuria, and polydipsia, and also cramps, blurred vision, and a major susceptibility to infection. Patients affected by type I diabetes have an increased incidence of microvascular complications and microvascular diseases [4]. They can also manifest diabetic ketoacidosis, characterized by an acetone odor of the breath and a general situation of dehydration [5].

High blood glucose levels may affect the heart and the blood vessels, and also kidneys, nerves, and eyes. The most common long-term complications are diabetic retinopathy (PDR) and peripheral neuropathy, which affects the nervous system and consists of a breakdown of the pain sensation in the extremities [6].

In addition, diabetic pathology causes an alteration of bone metabolism, a reduction in the percentage of bone formation, and an alteration of its remodeling. All the sequelae affecting the skeletal system fall under the name of diabetic bone disease or diabetic osteopathy [7].

Clinical and in vivo studies have shown impaired intramembranous and endochondral ossification in patients with diabetes [8].

Diabetes mellitus is closely involved in several systemic and oral manifestations, and evidence from the literature indicates that early implant failure is reported in patients with diabetes. The oral manifestations and complications related to DM include dry mouth (xerostomia), gingivitis, and periodontal disease. The intensity of the complications depends on the degree and duration of the hyperglycemia [9,10].

Diabetes is also responsible for an increased risk of tooth loss [11], bone loss, and periodontal tissue destruction; the link between diabetes and periodontitis can be attributed to various factors. Firstly, uncontrolled diabetes can lead to elevated blood sugar levels, which can impair the body’s ability to fight off infections, including those affecting the gums; this weakened immune response makes individuals with diabetes more susceptible to periodontal bacteria and their damaging effects on the gum tissue [12].

Moreover, periodontitis can contribute to poor glycemic control in diabetic patients. The inflammation associated with gum disease can increase insulin resistance, making it more challenging for the body to regulate blood sugar levels effectively. Additionally, the chronic low-grade infection and systemic inflammation caused by periodontitis can worsen insulin resistance and glycemic control [9]. Many studies have investigated the effects of diabetes on the response to periodontal therapy. The presence of diabetes increases the risk of recurrence and, at the same time, reduces the effectiveness and success of therapies. The worse the glycemic control of diabetes is, the less effective the therapies [13].

According to the literature, the effectiveness of implant treatment in patients with controlled diabetes is controversial, but a high success rate is reported by Fiorellini et al., [14] even though early implant failure is more common in diabetic patients. Furthermore, diabetic patients have also a higher rate of peri-implant mucositis and peri-implantitis than non-diabetic patients [15].

Nowadays, dental implants can be considered feasible procedures for oral rehabilitation in controlled diabetic patients, while patients with poorly controlled diabetes may undergo several complications after implant placement [16].

Certain conditions must be met for implant rehabilitation to be performed following the prosthetic plan. First of all, a sufficient amount of bone is necessary, both horizontally and vertically. It is well-known that the alveolar process goes through a significant resorption process after tooth loss, therefore restoring new bone might be needed [17]. Among the various reconstruction methods for treating potential dental implant sites with bone defects is guided bone regeneration (GBR): the GBR procedure involves the use of a barrier membrane, typically made of biocompatible materials such as resorbable or non-resorbable membranes, which is placed over the bony defect. This membrane acts as a physical barrier, preventing the migration of non-osteogenic cells such as epithelial cells into the defect area, while allowing the migration and proliferation of osteogenic cells. To enhance the regenerative process, bone graft material, such as autografts (patient’s own bone), allografts (donor’s bone), xenografts (animal bone), or synthetic bone substitutes, is used in conjunction with the barrier membrane. This graft material provides a scaffold for new bone formation and supports the growth of osteoblasts, the cells responsible for bone synthesis [18,19].

Diabetes and hyperglicemia effects on wound healing processes are well known, therefore it is expected that they could also have a negative effect on GBR, especially since uncontrolled diabetes is closely related to an increased prevalence of infective complications and less predictable outcomes in terms of bone regeneration [20].

Studies conducted on an animal model show that significant de novo bone formation can be reached with GBR, even in the case of uncontrolled diabetes [21].

However, further studies are needed to confirm these findings and to therefore conclude if bone regeneration after GBR procedures is affected by diabetes and hyperglicemia [22].

This study aims to assess the prognostic significance of the glycated hemoglobin (HbA1c) levels in patients undergoing horizontal GBR before implant placement and the correlation between different glycaemic control levels and clinical findings. The null hypothesis is that Glycated hemoglobin levels have no effects on the success of GBR procedures.

## 2. Materials and Methods

Subjects were recruited from patients requiring implant placement that had undergone horizontal guided bone regeneration at the department of oral surgery and implantology of the Catholic University of the Sacred Heart and La Sapienza University, Rome, Italy. All reported investigations were carried out in accordance with the 1975 Helsinki Declaration, as revised in 2013, for ethical approval. This prospective clinical study was approved by the local ethics committee with protocol code 0000110, 21 January 2020, and all participants provided written informed consent after receiving all relevant information regarding the study objectives and procedures. A single trained and experienced surgeon performed all surgeries, and the same examiner recorded all the clinical measures.

The inclusion criteria are:

- in need of one or more implants in the upper or lower jaw;
- in need of horizontal bone augmentation;
- Full-mouth plaque score (FMPS) and full-mouth bleeding score (FMBS) < 15%;
- age > 20 years.

Patients were excluded from the study if they had any of the following contraindications:

1. General contraindications for receiving dental implants or undergoing surgical treatment.
2. Uncontrolled periodontal disease.
3. Use of medications known to impact oral health and bone turnover, or that are contraindicated for surgical procedures, such as immunosuppressants, corticosteroids, or bisphosphonates.
4. Previous history of cancer, radiation therapy, or chemotherapy for cancer treatment.
5. Active smoking.
6. Blood-related disorders.
7. Limited mental capacity, language skills, or known psychological disorders.
8. Excessive alcohol consumption.
9. Conditions that affect the relationship between HbA1c (glycated hemoglobin) and blood sugar levels, such as sickle cell disease, pregnancy, glucose-6-phosphate dehydrogenase deficiency, HIV, hemodialysis, recent blood loss or transfusion, or use of erythropoietin therapy.
10. Unwillingness to return for scheduled follow-up examinations.

An assessment was carried out to obtain the patient’s age, sex, race, oral hygiene maintenance therapy (yes: at least one prophylaxis per year/no: less than one per year or none), previous periodontal therapy (yes: periodontal therapy before surgery/no: no periodontal therapy before surgery), obesity (normal/overweight/obese), osteopenia/osteoporosis (yes/no), mouthwash use (yes/no) and interdental hygiene (yes/no).

Blood glucose levels were determined preoperatively by HbA1c. Patients were divided into three groups:

- non-diabetic normoglycemic patients (HbA1c < 5.7%)
- non-diabetic hyperglycaemic patients (HbA1c < 6.5%)
- Patients with controlled diabetes (HbA1c < 7%)

Patients with non-controlled diabetes (HbA1c > 7%) were excluded from the present study.

The body mass index (BMI) of participants in all groups was calculated by estimating the weight in kilograms (kg) and height in meters squared (m^2^). Each patient’s pharmacological therapy has been recorded.

The baseline findings were considered before the surgical procedure. Every patient underwent radiological examinations before surgery to complete preoperative planning. A preoperative cone-beam computed tomography (CBCT) scan was performed so that the bone height and thickness of the cortical plates could be evaluated preoperatively.

The primary outcomes were the horizontal (mm) and vertical (mm) dimensional changes of the alveolar ridge at 6 months after the procedure. A CBCT scan was taken at 6 months of healing with a resolution of 100 µm (Orthophos XG 3D, Dentsply Sirona, Charlotte, NC, USA).

During the follow-up period, the researchers documented any negative incidents such as wound infection, graft exposure, soft tissue separation, and necrosis. The progress of wound healing was evaluated using the Early Wound Healing Score (EHS), which consists of three components: observable signs of re-epithelization, signs of hemostasis, and signs of inflammation. The total score of these three components determines the EHS. An optimal wound healing outcome is indicated by a score of 10, while the worst possible score is 0. The researchers assessed the EHS every seven days for the initial three weeks.

Before surgery, patients received antibiotics (2 × 1 g amoxicillin clavulanate). A povidone-iodine solution swab with sterile gauze, mounted in a Klemmer forceps, was used to disinfect the perioral skin. Patients were covered with TNT drapes, and the oral cavity was left uncovered. A gauze soaked in 0.2% chlorhexidine was used to clean the mucous membranes.

The surgery was conducted using local anesthesia, specifically articaine 4% with epinephrine 1:100,000. A horizontal crestal incision was made in the lower jaw and slightly buccal on the upper jaw, starting from the distal aspect of the mesial tooth and reaching the mesial aspect of the distal tooth. The incision was continued intrasulcularly in both the buccal and lingual areas. Releasing incisions were performed at the buccal, mesial, and distal line angles. A mucoperiosteal flap was elevated, and the bone was exposed and carefully curetted. Periosteal-releasing incisions were used to allow tension-free adaptation of the mucoperiosteal flaps. The adjacent teeth were carefully cleaned by ultrasonic and manual instruments. The defect was measured using a periodontal probe. A round bur was used to perforate the cortical plate in order to increase bleeding and therefore facilitate graft integration.

A resorbable membrane was custom-shaped to match the specific area where it was needed and secured using two or three fixation pins on the lingual/palatal side. Autogenous bone chips were collected using a bone scraper and mixed with deproteinized bovine bone in equal parts (50:50 ratio) to completely fill the defect. The membrane was then closed over the graft and secured on the buccal side using two or three fixation pins. The original horizontal incision was stitched using mattress sutures, and additional single sutures were placed along the vertical incisions and between the mattress sutures.

The patients were advised to rinse their mouths twice a day using a mouth rinse containing 0.2% chlorhexidine. They were also instructed to continue taking the prescribed antibiotics for a duration of five days. Furthermore, they were provided with analgesics to manage pain for the following three days, with the dosage of 2–3 tablets of 600 mg ibuprofen tailored to their individual requirements. Patients were also instructed to refrain from mechanical plaque removal in the area for 2 weeks. Sutures were removed 21 days following surgery.

The dental implants were placed approximately 6 to 9 months after the bone regeneration procedure. The implants had varying lengths ranging from 8 to 12 mm and diameters ranging from 3.3 to 5 mm. The implant placement procedure followed the recommended protocol provided by the manufacturing companies. To determine the primary stability of the implants, the adequacy of insertion torque and manual testing were used as assessment criteria.

A descriptive analysis was conducted for the demographic and clinical characteristics of the patients. Values are expressed as the mean and standard deviation for continuous variables and as frequency and percentage for categorical variables. Comparisons between categorical variables were made using the Chi-square test or Fisher’s test, depending on the circumstances.

The Kruskal–Wallis non-parametric test was conducted to check if there were statistically significant differences between the three groups taken as a whole. In addition, the Mann–Whitney test was performed to investigate the relationship between the three groups, comparing pairs of all groups to each other. Statistical analysis was conducted using IBM SPSS software, version 25.

## 3. Results

The study sample consisted of 54 patients (28 women and 26 men), aged between 48 and 80, with a mean of 66.44 years and a standard deviation of 8.02 years. Group 1 included 19 patients (mean age: 65.84 ± 8.64 years), 17 patients (mean age: 65.24 ± 8.67 years) were included in group 2, and 18 patients (mean age: 68.22 ± 6.75 years) were included in group 3. The three groups did not show statistically significant differences in the age (*p* = 0.635) and sex variables (*p* = 0.981).

All patients underwent horizontal bone regeneration procedures between 1 February 2020 and 1 July 2020. In total, 71 dental implants were placed. A single implant was placed in 37 participants (68.5%), and 17 patients (31.5%) received two implants. All surgeries were performed successfully, no intraoperative complications were recorded.

The complications observed consisted of wound dehiscence and consequent exposure of the membrane, subsequently treated with local disinfection or with rinsing with 0.2% chlorhexidine-based mouthwash and application of 1% chlorhexidine gel. These episodes occurred in patients who received 3.3 mm implants and, in these cases, delayed healing of the surgical site was recorded.

The overall mean value of the vertical defect at baseline was 0.50 ± 0.69 mm (Table 1), while the horizontal defect had an average value of 2.98 ± 0.73 mm (Table 2).

The mean vertical defect in group 1 was 0.58 ± 0.77 mm; in group 2, it was 0.47 ± 0.71 mm; and in group 3, it was 0.44 ± 0.61 mm. The distribution of the vertical defect was the same within the three groups, with a *p*-value of 0.881. The respective vertical gains averaged 0.53 ± 0.77 mm in group 1, 0.53 ± 0.62 mm in group 2, and 0.44 ± 0.61 mm in group 3, with no statistically significant differences between groups (*p* = 0.904), with an overall mean value of 0.50 ± 0.67 mm (Table 3).

The mean of horizontal defects in group 1 was 3.21 ± 0.85 mm; in group 2, it was 2.94 ± 0.66 mm; and in group 3, it was 2.78 ± 0.65 mm. Also, the groups were homogeneous for the considered variable in this case, with a *p*-value of 0.243, which was not statistically significant. A statistically significant horizontal bone gain was recorded after the GBR (*p* < 0.05) in all groups. The respective horizontal gains presented an average value of 3.47 ± 0.77 mm, 2.94 ± 0.55 mm, and 3 ± 0.69 mm, respectively; the overall horizontal gain averaged 3.15 ± 0.71 mm (Table 4).

There was a statistically significant difference between the three groups in terms of horizontal gain at 6 months: in particular, there was a statistically significant difference between group 1, consisting of non-diabetic normoglycemic patients, and group 2, comprising non-diabetic hyperglycemic subjects (*p* = 0.026) and between group 1 and group 3 (*p* = 0.030). In contrast, there were no statistically significant differences between group 2 and group 3 (*p* = 0.930)

All implants achieved adequate primary stability during the implant placement surgery. Sixty-eight implants (95.8%) were classified as “successful” and three implants (4.2%) as “unsuccessful,” meaning the possibility of inserting a standard-sized implant with a diameter of ≥ 4 mm following the horizontal GBR procedure.

A post-hoc power analysis was run keeping the horizontal bone gain as the value of reference, with a type 1 error rate of 0.05 showed a power of 0.87.

## 4. Discussion

Diabetes mellitus has been growing rapidly in recent years, and the worldwide incidence of this disease has increased [23]. Diabetes predominantly affects the adult population. For this reason, the clinician must pay particular attention to this trend, as it is especially the adult and older patient who requires implant-prosthetic rehabilitation.

Although bone metabolism appears to be altered in subjects with diabetes mellitus, a systematic review by Chrcanovic et al. reported no significant difference in terms of implant failure between diabetic and non-diabetic patients [24]. Therefore, it is possible to place dental implants in diabetic patients, obtaining favorable outcomes, if the glycemic status is within the controlled range.

However, the risks in the case of uncontrolled diabetes range from increased healing times to the development of serious infections that slow the process of osseointegration of the implant. Sometimes, unscheduled bone dehiscences or fenestrations may occur during normal osteotomy site preparation or implant insertion, requiring a bone augmentation procedure to avoid leaving the implant surface exposed.

Diabetic bone is characterized by a reduced turnover, in which there is a prevalence of the bone resorption process over that of neo-apposition, resulting in reduced bone mineral density, greater tendency to fracture [25], poor bone healing, and impaired bone regeneration potential [26].

Currently, the pathophysiology of the cellular mechanism in impaired diabetic bone healing is not fully understood. Additionally, impaired intramembranous bone healing was observed in the early stages of healing in animal models with experimental type 1 diabetes [27].

Furthermore, diabetes appears to have adverse effects in terms of bone formation and osseointegration during GBR procedures.

The osseointegration process between bone and implant in the patient with diabetes may be sometimes compromised due to the effect of chronic hyperglycemia on bone mineralization and remodeling, with consequent reduced BIC (bone-implant contact) [28].

Bone-Implant Contact (BIC) refers to the direct interface between an implanted medical device, such as a dental implant or orthopedic implant, and the surrounding bone tissue. It is a critical factor for the success and long-term stability of the implant.

However, it appears that good glycemic control, also based on the administration of insulin, improves osseointegration and implant survival. At the same time, some studies find less BIC in diabetic subjects than in non-diabetic subjects [14].

Six months after the GBR procedure, the present study found a statistically significant difference in horizontal bone gain between group 1, composed of normoglycemic non-diabetic patients, and group 2, with hyperglycemic non-diabetic patients. According to Tawil, the different glycemic level represents a possible risk factor for implant and regenerative surgery procedures [29].

Glycated hemoglobin was adopted for monitoring glycemic control in these diabetic patients given its capabilities of reflecting the average blood glucose levels over the preceding two to three months. The HbA1c value is expressed as a percentage, representing the proportion of glycated hemoglobin to total hemoglobin in the blood. The American Diabetes Association (ADA) recommends an HbA1c target of less than 7% for most individuals with diabetes, although individualized targets may vary based on factors such as age, comorbidities, and personal circumstances. Regular monitoring of HbA1c is essential for individuals with diabetes to assess the effectiveness of treatment, identify the need for adjustments in medication or lifestyle, and mitigate the risk of complications associated with poorly controlled blood sugar levels.

There was a greater horizontal bone gain of 3.47 ± 0.77 mm in normoglycemic subjects (HbA1c < 5.7%), compared to hyperglycemic patients (HbA1c < 6.5%), who reported a mean horizontal gain of 2.94 ± 0.55 mm and diabetic subjects (HbA1c < 7%) with 3 ± 0.69 mm.

The results of this study lead us to assume that the best clinical outcomes concerning this variable occurred in patients with lower HbA1c levels.

Erdogan et al. instead found that there are no statistically significant differences in terms of horizontal bone gain between diabetic and non-diabetic patients [30].

Despite the presence of controlled diabetes, with HbA1c < 7%, in group 3, it was possible to observe a slight vertical gain (0.44 ± 0.61 mm) at 6 months after surgery and a significant horizontal gain (3 ± 0.69 mm).

In this work, standard-sized implants with a diameter > 4 mm were placed following the GBR procedure in 95.8% of cases. This was not possible in only 4.2% of the cases, and implants with a diameter of 3.3 mm were placed instead.

These results are consistent with the evidence in the literature: a recent study conducted by Retzepi confirms the possibility of using GBR procedures to increase bone volumes in cases of deficient ridges, even in the presence of uncontrolled experimental diabetes [20].

Furthermore, the healing process and the incidence of complications improve as metabolic control increases, and the contact between newly formed diabetic bone tissue after GBR and implant are similar to the contact between healthy bone and implant [31].

The research group led by Hasegawa established that diabetes mellitus did not influence this parameter [32].

The main complication observed in this work was the membrane exposure in patients who had 3.3 mm implants inserted.

The literature shows that the most frequent long-term complication in patients with diabetes is peri-implantitis, which arises a few years after the prosthetic load. It results from the increased susceptibility to infections and the reduced host immune response to bacteria shown by these patients [33].

The main limitations present within this study are related to the small sample of participants and a short-term follow-up. Additional data are useful for understanding the relationship between diabetes mellitus and the clinical outcomes of regenerative surgery could be obtained by analyzing cellular or molecular factors such as osteoclasts, osteoblasts, and biological markers to clarify the mechanism of impaired bone healing as well as histological analysis to assess the features of the regenerated bone. Furthermore, long-term studies are needed to assess the eventuality of late complications.

## 5. Conclusions

GBR before implant placement is a feasible procedure for treating horizontal bone defects in patients with controlled type 2 diabetes.

The present investigation shows that patients with HbA1c levels below 7% can undergo GBR procedures and implant placement, obtaining a statistically significant horizontal bone gain.

A larger-scale and long-term study with histological analysis should be conducted to confirm the findings emerging from the present study.

## Figures and Tables

**Table 1:** Patients’ preoperative vertical bone defect.

Group	Vertical Defect (Mean ± SD)	
1	0.58 ± 0.77 mm	*p* = 0.881
2	0.47 ± 0.71 mm	
3	0.44 ± 0.61 mm	
Overall	0.50 ± 0.69 mm	

**Table 2:** Patients’ preoperative horizontal bone defect.

Group	Horizontal Defect (Mean ± SD)	
1	3.21 ± 0.85 mm	*p* = 0.243
2	2.94 ± 0.66 mm	
3	2.78 ± 0.65 mm	
Overall	2.98 ± 0.73 mm	

**Table 3:** Patients’ postoperative vertical bone gain.

Group	Vertical Gain (Mean ± SD)	
1	0.53 ± 0.77 mm	*p* = 0.904
2	0.53 ± 0.62 mm	
3	0.44 ± 0.61 mm	
Overall	0.50 ± 0.67 mm	

**Table 4:** Patients’ postoperative horizontal bone gain.

Group	Horizontal Gain (Mean ± SD)	
1	3.47 ± 0.77 mm	*p* = 0.037
2	2.94 ± 0.55 mm	
3	3 ± 0.69 mm	
Overall	3.15 ± 0.71 mm	

## Data Availability

Data is available on request to the corresponding author.
